# Adaptation to recent conflict in the classical color-word Stroop-task mainly involves facilitation of processing of task-relevant information

**DOI:** 10.3389/fnhum.2015.00088

**Published:** 2015-03-03

**Authors:** Sascha Purmann, Stefan Pollmann

**Affiliations:** ^1^Experimental Psychology, Department of Psychology, Otto-von-Guericke UniversityMagdeburg, Germany; ^2^Center for Behavioral Brain SciencesMagdeburg, Germany

**Keywords:** fMRI, color-word Stroop, conflict adaptation, VWFA, V4, cognitive control

## Abstract

To process information selectively and to continuously fine-tune selectivity of information processing are important abilities for successful goal-directed behavior. One phenomenon thought to represent this fine-tuning are conflict adaptation effects in interference tasks, i.e., reduction of interference after an incompatible trial and when incompatible trials are frequent. The neurocognitive mechanisms of these effects are currently only partly understood and results from brainimaging studies so far are mixed. In our study we validate and extend recent findings by examining adaption to recent conflict in the classical Stroop task using functional magnetic resonance imaging. Consistent with previous research we found increased activity in a fronto-parietal network comprising the medial prefrontal cortex, ventro-lateral prefrontal cortex, and posterior parietal cortex when contrasting incompatible with compatible trials. These areas have been associated with attentional processes and might reflect increased cognitive conflict and resolution thereof during incompatible trials. While carefully controlling for non-attentional sequential effects we found smaller Stroop interference after an incompatible trial (conflict adaptation effect). These behavioral conflict adaptation effects were accompanied by changes in activity in visual color-selective areas (V4, V4α), while there was no modulation by previous trial compatibility in a visual word-selective area (VWFA). Our results provide further evidence for the notion, that adaptation to recent conflict seems to be based mainly on enhancement of processing of the task-relevant information.

## 1. Introduction

While our senses are able to process an enormous amount of information, we are unable to process all the information that is available in the environment at any point in time. Given this limitation, for successful goal-directed behavior our information processing system has to be selective: It has to process goal-relevant information with higher priority than irrelevant information. Interestingly, in everyday life and during performance of tasks used in laboratory research our cognitive system seems to move along a continuum from more to less selective information processing (Durstewitz and Seamans, 2008; Diamond, 2013). The selectivity of information processing seems to be adjusted in accordance with situation/task context and this fine-tuning can occur on a short temporal scale of a couple of milliseconds to seconds (Logan and Zbrodoff, 1979; Gratton et al., 1992; Kerns et al., 2004).

Given the importance of these processes for everyday life, it is no surprise to find selective attention and control thereof as a main area of research in cognitive psychology and cognitive neuroscience (for recent reviews see Banich, 2009; Hofmann et al., 2012). The tasks typically used to study selective attention in the laboratory involve discrimination of a stimulus or dimension of a stimulus while ignoring other stimuli or dimensions of a stimulus. For instance, subjects might have to indicate the identity of a letter while ignoring distractor letters on the left and right of the target stimulus (Eriksen flanker task; Eriksen and Eriksen, 1974) or to indicate the color of a color word while ignoring its word meaning (Stroop color-word task; Stroop, 1935).

In terms of error rates participants' performance in these tasks is normally very good, showing that they are able to give task-relevant information higher priority in information processing than task-irrelevant information. Nevertheless, task-irrelevant information is not filtered out completely but processed to a certain degree as well. This is reflected in slower responses and more frequent errors for stimuli for which the task-relevant information and the task-irrelevant information is associated with different responses. For example, in the Stroop task, naming the color (e.g., green) of an incompatible color word like RED is slower and more error prone than naming the color of a compatible target word GREEN (for reviews see MacLeod, 1991; MacLeod and MacDonald, 2000).

Interestingly, this interference effect gets weaker directly after an incompatible trial (Gratton et al., 1992; Kerns et al., 2004). Current models of cognitive control account for this finding by assuming that attentional adjustment, i.e., fine tuning of selectivity of information processing, occurs in response to cognitive conflict. One account assumes that the main mechanism of conflict adaptation involves a later stage in information processing (adjustment of response threshold; Gratton et al., 1992). Another account assumes that adaptation to recent conflict is realized by enhancement of processing of task-relevant information early in information processing (Botvinick et al., 2001). Similar behavioral effects have been shown for trials following error trials, i.e., slower (but not necessarily more accurate) responses following error trials (Rabbitt and Rodgers, 1977), probably reflecting increased response caution (Dutilh et al., 2012) and reduced interference in interference tasks in trials following error trials (King et al., 2010).

While these models propose specific mechanisms it is important to note that cognitive control in these instances could be implemented in various ways: At the sensory level processing of task-relevant sensory information could get facilitated and/or processing of task-irrelevant sensory information could get suppressed. Furthermore, stimulus-response translation could be altered or, at the motor level, the response threshold could be increased. Finally, any mixture of these mechanisms seems possible. More globally, these adjustments seem to be accompanied by changes in representation of information higher-level areas. It has been shown that neurons in the frontal and parietal lobes represent stimulus features in an adaptive way based on current task demands (Woolgar et al., 2011).

Whether inhibition is one of the mechanisms of cognitive control is currently still highly debated. Some authors argue that top-down inhibition is biologically implausible, as inhibitory connections in the human brain are strictly local (Herd et al., 2006). However, while inhibition plays a key role for information processing in small neural networks, GABAergic (i.e., inhibitory) projection neurons have been found in the brain as well (originating in the septum region, the hippocampus and the neocortex; Tamamaki and Tomioka, 2010). Additionally, glutamatergic (i.e., excitatory) projection neurons could result in inhibition of a brain area when synapsing on local inhibitory neurons. Given these two arguments, we do not see biological reasons to exclude long-range inhibition as a mechanism a-priori.

Importantly, in most cases, the behavioral effects attributable to cognitive control can be accounted for equally well with and without an inhibitory mechanism (MacLeod et al., 2003; Egner and Hirsch, 2005). Therefore, a prominent strategy used to pin down the exact mechanisms of conflict adaptation makes use of cognitive neuroscience methods such as event-related brain potentials (ERPs) or functional magnetic resonance imaging (fMRI). Certain ERPs and BOLD activity in certain brain regions of interest can be used as indicators for certain cognitive processes.

Whether adaptation to recent conflict is mediated by enhancement of processing of task-relevant information, suppression of processing of task-irrelevant information, or by both has been examined explicitly in an fMRI study using this strategy (Egner and Hirsch, 2005). In this study evidence for enhancement of processing of task-relevant information only was found. This result is interesting, as another fMRI study on task-set implementation in the Stroop task has found enhancement of task-relevant information and suppression of task-relevant information (Polk et al., 2008). Importantly, while the first study has to be interpreted carefully because it uses a face-word Stroop task with non-integrated stimuli and task-switching between blocks, the second task is difficult to interpret as transient and sustained effects cannot be disentangled because of the use of a block design. With our study we were aiming at further clarifying the neural mechanisms underlying conflict adaptation.

While participants performed a color-word Stroop task (Stroop, 1935) we measured the blood-oxygenation level dependent (BOLD) response using magnetic resonance imaging. The Stroop task is one of the interference tasks most often used to study selective attention and cognitive control in the laboratory (for a review see MacLeod, 1991), so the neural underpinnings of cognitive processes involved in this task will be of interest to a broad readership. To examine if adaptation to recent conflict involves enhancement or suppression of sensory information processing, we identified inferotemporal brain regions involved in color (visual areas V4 and V4α; cf. Bartels and Zeki, 2000) and word processing (visual word form area/VWFA; cf. Cohen et al., 2000; Reinholz and Pollmann, 2005; but see also Price and Devlin, 2003) in an independent localizer task. We used activity in these relatively well-understood, functionally defined brain regions as indicators for early sensory information processing of task-relevant and task-irrelevant information, respectively. Increases of activity in V4/V4α after a conflict trial would lend support for an enhancement model, a reduction of activity in VWFA would support a suppression model, while a combination of activation increase in V4 and reduction of activity in VWFA would support a dual-mechanism model. No modulation of sensory brain areas while seeing conflict adaptation effects in behavior would speak for modulation at later processing stages such as changing stimulus-response translation or adjustment of the response-threshold of the motor system.

When investigating adaptation to recent conflict, it is crucial to control for the sequence of stimulus features (e.g., colors and words) and responses. In standard paradigms (i.e., using limited sets of stimuli and responses), repetitions of the compatibility level (i.e., a compatible trial following a compatible trial or an incompatible trial following an incompatible trial) tends to be associated with either repetition or alternation of both the target and the distractor information. Compatibility level alternations, on the other hand, tend to be associated with repetition of either the target or the distractor information and alternation of the other (i.e., partial feature repetition). According to event file theory (Hommel, 2004), partial feature repetitions are associated with a processing disadvantage because of a mismatch between the prior processing episode and the current task demands. It is therefore possible that reductions of interference effects after conflict trials reflect such processing disadvantages rather than an adjustment of selectivity of information processing (Notebaert et al., 2006). To control for feature-integration effects, we applied a 6:6 mapping between colors and responses and created pseudo-random stimulus-sequences that only included complete alternations of stimulus features from one trial to the next. More specifically, no color reoccurred as color or word on the next trial and no word reoccurred as color or word on the next trial. Another method to deconfound feature-integration and conflict adaptation effects is to exclude partial and/or complete repetitions from the analysis (e.g., Kerns et al., 2004) but lead to a substantial loss of data. Importantly, feature-integration and conflict adaptation effects has successfully been deconfounded by the described methods (for an example in the Eriksen flanker task see Wendt et al., 2014).

## 2. Materials and methods

### 2.1. Participants

We recruited 20 students from the population of students of the University of Magdeburg. Data from two participants had to be excluded from the analysis, one because of an imaging artifact and one because of technical problems in collecting the behavioral data, resulting in a final sample of 18 students (8 male; age range 20–29; mean age: 24). Vision of all participants was normal or corrected to normal and none of the participants reported any neurological or psychiatric abnormality or conditions contraindicating MRI. Additionally, by self report none of the participants was color blind, all participants had a right hand preference and were native German speakers. Participants were paid or participated for partial course fulfillment. The experiment was carried out in accordance with the Declaration of Helsinki.

### 2.2. Experimental paradigms

Participants performed three different tasks: One task used to localize visual areas relevant for color processing, one task used to localize brain areas relevant for word processing and a color word Stroop task (Stroop, 1935).

Color localizer task. Participants performed a task similar to the Farnsworth-Munsell 100-Hue Test (Beauchamp et al., 1999). This task has been found to reliably activate inferotemporal brain regions related to color processing. Participants saw a series of displays that were block-wise either achromatic (non-color condition) or chromatic (color condition). Each display was composed of an array of five wedges arranged in a circular fashion around a fixation cross presented at the center of the screen. These wedges could form a continuous sequence or include one wedge that did not fit in. Participants had to decide, if the sequence was continuous or not and give their answer by pressing a button with their right or left index finger, respectively.

Word localizer task. Participants performed a one-back memory task. Four-letter words with comparable frequency (Institutfür Deutsche Sprache, 2009); word condition) and four-digit numbers (non-word condition) were used as stimuli (c.p. Park et al., 2012). Participants saw a series of either words or numbers in a block-wise fashion and had to indicate any direct repetition of a word (or number) by pressing a button with their right index finger and a change by pressing a button with their left index finger.

Stroop task. Color words (blue, red, green, yellow, orange, violet) printed in different colors (blue, red, green, yellow, orange, violet) were presented above a fixation mark. Participants had to indicate the font color by pressing one of six buttons. For ease of task performance, response-to-button mappings were presented at the bottom part of the screen, and participants were trained prior to the fMRI session. Participants used the index, middle and ring fingers of their right and left hands for responding. Response-to-button mappings were randomly assigned to participants. In compatible trials, word meaning and word color were the same. In incompatible trials, word meaning and word color differed. Compatible and incompatible trials were presented with equal probability.

For all tasks, participants were instructed to keep their eyes on the fixation cross and to respond as fast as possible while trying to keep the error rate between five and ten percent. Mean response time and error rate were provided as feedback after each Stroop fMRI run.

Overall, participants performed seven runs of 7–8 min each: Two color localizer runs, two word localizer runs, and three Stroop runs. During a word localizer run participants performed eight task blocks, 29 s in length. In each task block, 20 displays were presented, each for 500 ms and an inter-stimulus-interval of 1000 ms. There was a fixation interval of 20 s between blocks. During a color localizer run participants performed eight task blocks, 29 s in length. In each task block, 10 displays were presented, each for 2000 ms with an inter-stimulus-interval of 1000 ms. There was a fixation interval of 20 s between blocks. During a Stroop run, participants performed 100 trials. Trials were jittered with a mean inter-trial-interval of 2.5 s. At the beginning of each trial the fixation cross disappeared for 200 ms (warning signal), after another 300 ms the stimulus was presented for 400 ms. Each participant started with a localizer run (color or word localizer, randomized between participants), continued with a Stroop run, after which localizer and Stroop runs alternated.

### 2.3. Analysis of behavioral data

Median RTs (Ratcliff, 1993) and arcsine squareroot transformed error rates were analyzed using separate repeated-measures analyses of variance (ANOVA). All RT analyses excluded error trials and trials immediately following errors and trials, in which participants did not respond. Arcsine squareroot transformed error rates were used to normalize the data due to a positive skew frequently associated with error-rate data (Neter et al., 1985).

### 2.4. MRI data acquisition and analysis

Magnetic resonance imaging was performed on a 3T Siemens Trio MRI scanner with an 8-channel head coil. For functional imaging we used a T2^*^-weighted BOLD sensitive gradient echo echo-planar imaging (main task: TR = 2000 ms, TE = 32 ms, FA = 80, FOV = 19.2 cm, MAT = 64 × 64, 3 mm × 3 mm × 3 mm, 1 mm inter-slice gap, interleaved acquisition, 32 slices; localizer tasks: TR = 1500 ms, TE = 32 ms, FA = 80, FOV = 19.2 cm, MAT = 64 × 64, 3 mm × 3 mm × 3 mm, 1 mm inter-slice gap, interleaved acquisition, 24 slices). For the main task whole brain was covered, for the localizer task only the occipital, temporal and ventral frontal lobes were covered. The first 10 s of each run were discarded to allow for steady-state tissue magnetization. Prior to collection of functional data, T1-weighted anatomical images in the same plane as the functional images were acquired using a gradient-echo multi-slice sequence. High resolution T1-weighted Fast Low Angle SHot (FLASH; TR = 30 ms, TE = 4.4 ms, FA = 80, FOV = 19.2 cm, MAT = 64 × 64, 176 axial slices, resolution of 1 mm × 1 mm × 1 mm) anatomical images were collected at the end of the session to allow for localization and visualization of brain activation. Head motion was restricted using foam padding that surrounded the head. We back-projected the stimuli onto a screen, which was positioned behind the head coil. Subjects viewed this screen through a mirror attached to the head coil. Presentation® software (Neurobehavioral Systems, http://www.neurobs.com) was used to present stimuli and to collect responses.

#### 2.4.1. Preprocessing

All functional MRI analyses were carried out using FSL 5.0 (Smith et al., 2004). Images were corrected for slice time differences and small head movements (Jenkinson et al., 2002). Translational movement parameters never exceeded one voxel in any direction for any subject. During preprocessing, we applied spatial smoothing using Gaussian kernels of FWHM 6 mm as well as multiplicative mean intensity normalization of the volume at each time point and high pass temporal filtering (160 s).

#### 2.4.2. Univariate analysis

First Level Analysis. Localizer tasks. The localizer tasks resembled a block design and were analyzed accordingly. Two boxcar functions, each representing one condition (color vs. non-color, word vs. non-word, for the two localizer tasks respectively) were convolved with a double gamma function and fed as regressors into the general linear model (GLM). Additionally, six motion parameters estimated during preprocessing were included in the GLM as nuisance regressors. The GLM used a local autocorrelation correction (Woolrich et al., 2001).

***2.4.2.1. Stroop task***. The Stroop task resembled an event-related design and was analyzed accordingly. Within the general linear model framework, a model with four regressors of interest, one for each trial type (cC, cI, iC, iI; the lower case letter denotes conflict level of the previous trial and the upper case letter conflict level of the current trial), was calculated. Each regressor consisted of a series of impulse functions (50 ms), positioned at trial onset. These regressors were convolved with a double gamma function. Additionally, six motion parameters estimated during preprocessing, regressors for error trials and the first derivative of regressors of interest were included in the GLM as nuisance regressors. The GLM used a local autocorrelation correction (Woolrich et al., 2001).

***2.4.2.2. Second level and group analysis***. After statistical analysis for each single run, the resulting statistical images were normalized into common stereotactic space with isotropic voxels of 1 × 1 × 1 mm size, before the three runs of each participant were combined in subject-specific fixed-effects analyses. Results of this second level analysis were then fed into a random-effects group analysis. This analysis resulted in Z-statistic images. Normalization involved three steps: Registration of the average functional image to the low-resolution structural image, of the low-resolution structural image to the bias corrected high-resolution structural image, and of the bias corrected high-resolution structural image to the MNI T1 template. The different coregistration matrices were then combined to normalize statistical images resulting from the first level single subject analysis into MNI space. To correct for multiple comparisons in whole brain analyses we only retained clusters that exceeded a minimal size. These minimal cluster sizes were determined using Monte-Carlo simulations as implemented by AlphaSim (AFNI, http://afni.nimh.nih.gov/) and result in an overall *p* < 0.05 whole brain. Specific cluster sizes are given in the relevant parts.

#### 2.4.3. Identification of regions of interest

For each participant we identified four regions-of-interest (ROIs) for color processing restricted by anatomical and functional constraints. We created anatomical masks from a probabilistic atlas (Harvard-Oxford cortical structural atlas, Desikan et al., 2006) for the occipito-temporal fusiform gyrus (V4α) and the occipital fusiform gyrus (V4) for each hemisphere (c.p. Beauchamp et al., 1999; Bartels and Zeki, 2000) For each participant we then determined the peak voxel in the color vs. non-color contrast in these individual anatomical ROIs and calculated the median percent signal change for a sphere of 60 voxels (radius = 15 mm, volume = 2160 mm^3^) around these peak voxels.

For each participant we identified a ROI for word processing restricted by anatomical and functional constraints. We created anatomical masks from an probabilistic atlas (Harvard-Oxford cortical structural atlas, Desikan et al., 2006) for the posterior temporal fusiform gyrus (VWFA; c.p. Cohen et al., 2000; McCandliss et al., 2003; Reinholz and Pollmann, 2005). For each participant we then determined the peak voxel in the word vs. non-word contrast in these individual anatomical ROIs. We used a sphere of 60 voxel (radius = 15 mm, volume = 2160 mm^3^) around these peak voxels as our ROIs for the main analysis.

Because the inferotemporal cortex is prone to signal dropout effects due to its proximity to the ear canal, we also calculated temporal signal to noise ratio (TSNR) for these regions for each of the seven fMRI runs for each subject to ensure that the signal in our ROIs allows robust statistical analysis (Murphy et al., 2007).

## 3. Results

### 3.1. Behavioral results

**Figure 1:**
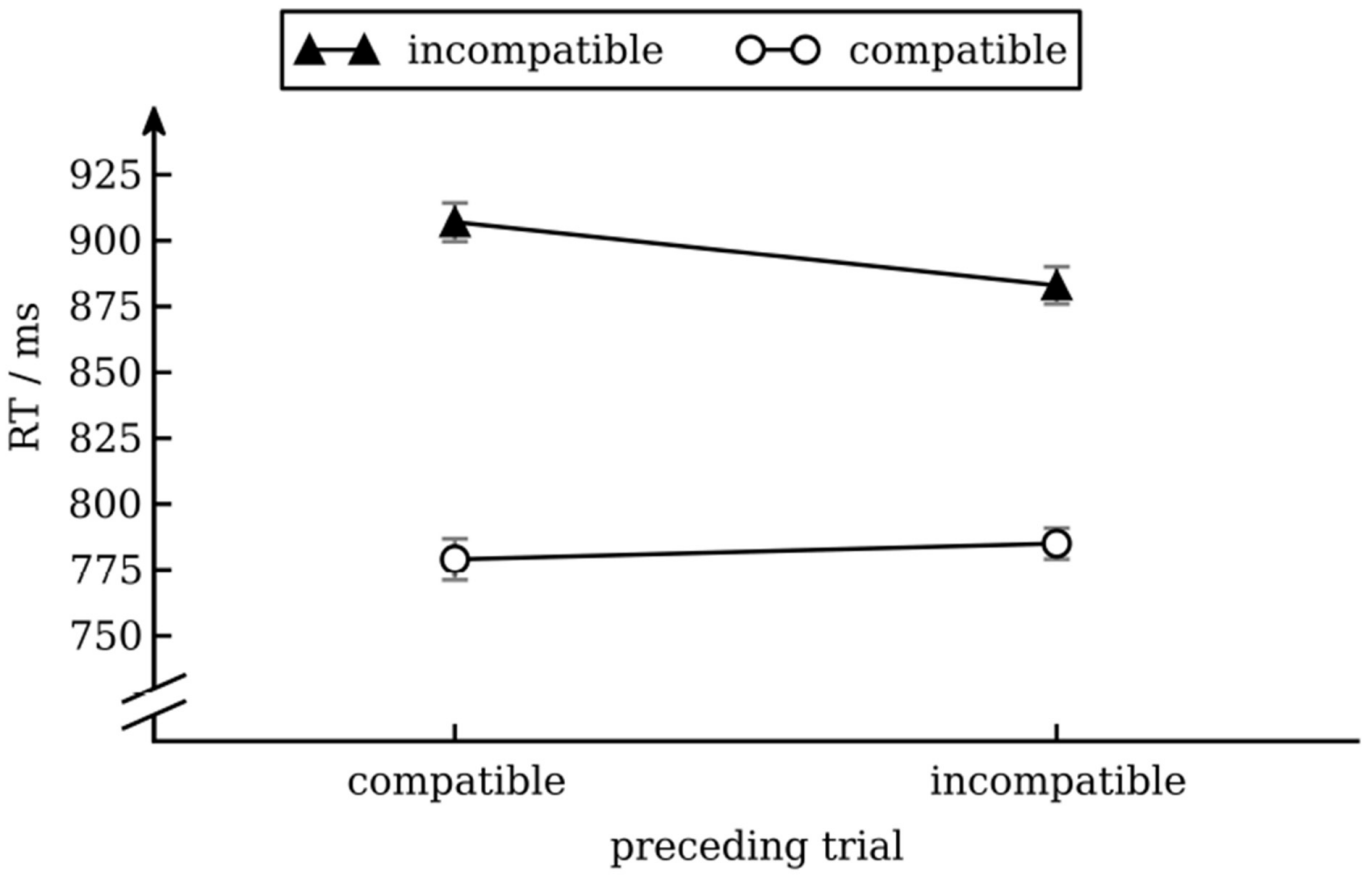
**Mean response times as a function of conflict in the previous and current trial**. Error bars show standard error of the mean (Cousineau and O'Brien, 2014).

We calculated a repeated measures ANOVA with median RT as dependent variable and conflict in the preceding trial (incompatible vs. compatible) and conflict in the current trial (incompatible vs. compatible) as independent variables. There was a main effect of conflict in the current trial [Stroop effect; *F*_(1, 17)_ = 101.9, *p* < 0.001], reflecting that overall response times were longer for incompatible (895 ms) than for compatible (782 ms) trials. There was also an interaction effect between conflict in the current trial and conflict in the previous trial [conflict adaptation; *F*_(1, 17)_ = 8.1, *p* < 0.05], reflecting that Stroop interference was smaller after an incompatible trial (98 ms) than after a compatible trial (128 ms). No other effects were significant. We present data in Figure 1.

**Figure 2:**
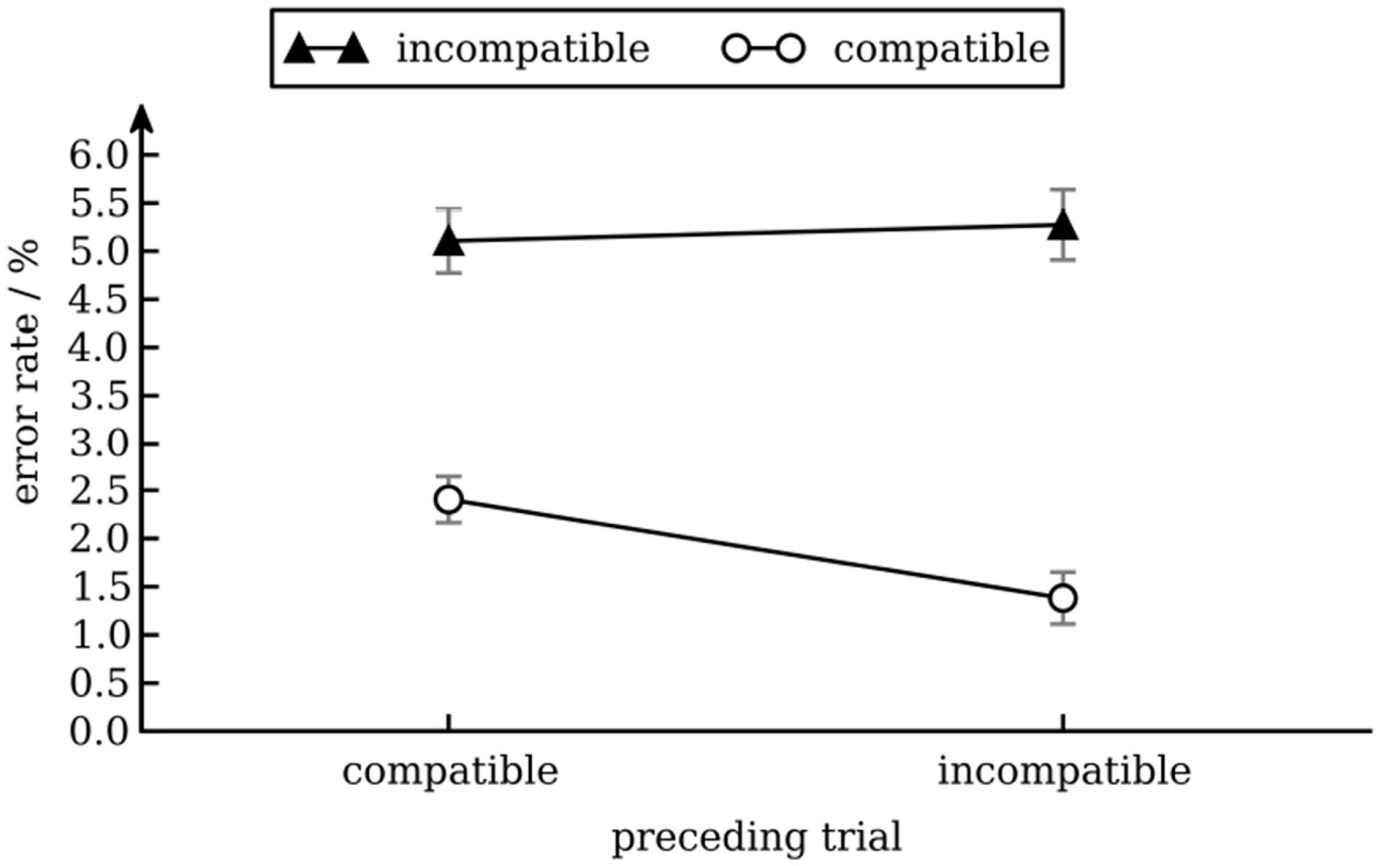
**Error rates as a function of conflict in the previous and current trial**. Error bars show standard error of the mean (Cousineau and O'Brien, 2014).

We calculated a repeated measures ANOVA with arcsine squareroot transformed error rates as dependent variable and conflict in the preceding trial (incompatible vs. compatible) and conflict in the current trial (incompatible vs. compatible) as independent variables. There was a main effect of conflict in the current trial [Stroop effect; *F*_(1, 17)_ = 29.0, *p* < 0.001], reflecting that error rates were higher for incompatible (5.2%) than for compatible trials (1.8%). No other effects were significant. Although there was a conflict adaptation effect in response times only, the pattern of error rates excludes speed-accuracy trade-off as a possible explanation for this pattern of response times (see Figure 2).

### 3.2. Neuroimaging results

**Figure 3:**
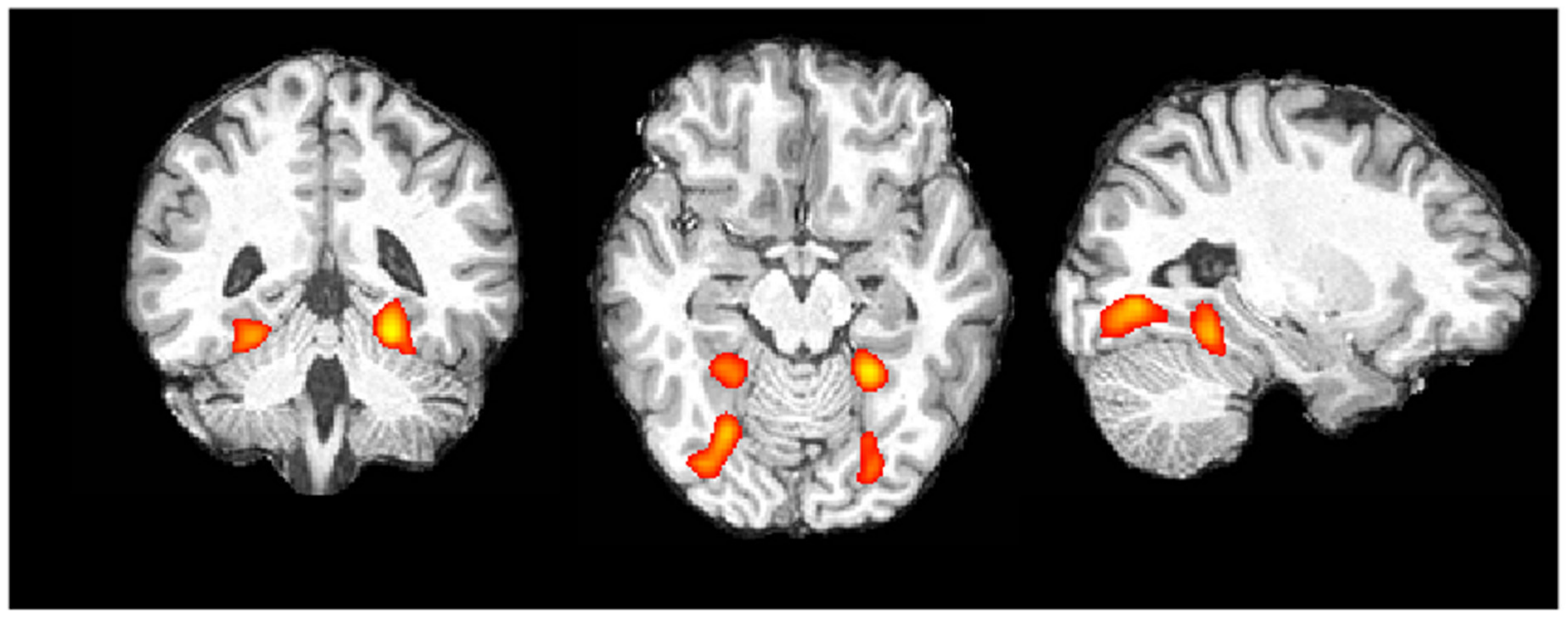
**Single subject data, color vs. non-color blocks (for display purposes thresholded at *z* > 6.0; image presented in radiologic convention)**.

Color Localizer. At the individual level all participants showed a clear pattern of activation. In each participant we found greater activation in an anterior and a posterior part of the fusiform gyrus in both hemispheres for color blocks compared to non-color blocks (see Figure 3 for an representative example). Data for peak voxels can be found in Table 1. Peak voxel coordinates are highly consistent with what has been found in other studies (c.p. Beauchamp et al., 1999; Bartels and Zeki, 2000).

**Table 1:** **Range of MNI coordinates and median coordinates for peak voxels of inferotemporal ROIs**.

**ROI**	**Hemisphere**	**Z_max_**	**x**	**y**	**z**	**TSNR**
V4	Left	3.64 to 9.67 (6.2)	−37 to −15 (−30)	−87 to − 61 (−73)	−19 to −5 (−11)	64
V4	Right	2.64 to 10.15 (6.3)	26 to 37 (31)	−81 to −61 (−70)	−17 to −5 (−11)	62
V4α	Left	2.98 to 10.22 (6.2)	−40 to −23 (−29)	−64 to −42 (−55)	−20 to −8 (−13)	57
V4α	Right	3.52 to 9.03 (6.1)	23 to 40 (29)	−58 to −37 (−47)	−26 to −8 (−17)	50
VWFA	Left	1.98 to 7.93 (4.2)	−46 to −21 (−39)	−45 to −11 (−32)	−33 to −15 (−20)	50

**Figure 4:**
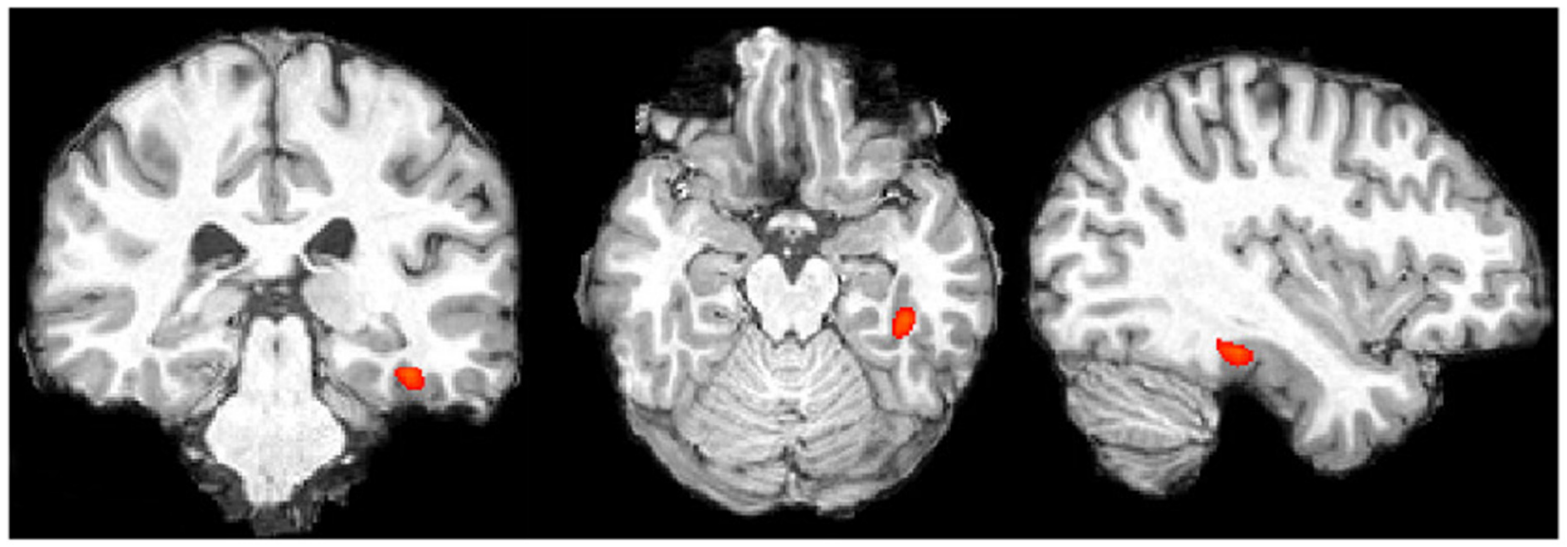
**Single subject data, words vs. number blocks (for display purposes thresholded at *z* > 6.0; image presented in radiologic convention)**.

Word Localizer. In each participant we determined the peak voxel in the posterior part of the temporal fusiform gyrus in the left hemisphere for word blocks compared to non-word blocks. Data for peak voxels can be found in Table 1 and data of a representative subject is presented in Figure 4. These coordinates are consistent with what has been found in other studies, although the activation in our study is more anterior to studies that contrasted letter string and pseudowords with similar complex symbol strings (c.p. Cohen et al., 2002; MNI x = 43, y = 54, z = 2). Nevertheless, our results replicate the localization that has been found in a recent study when contrasting letter strings with number strings (Park et al., 2012; MNI x = 36, y = 37, z = 23).

**Figure 5:**
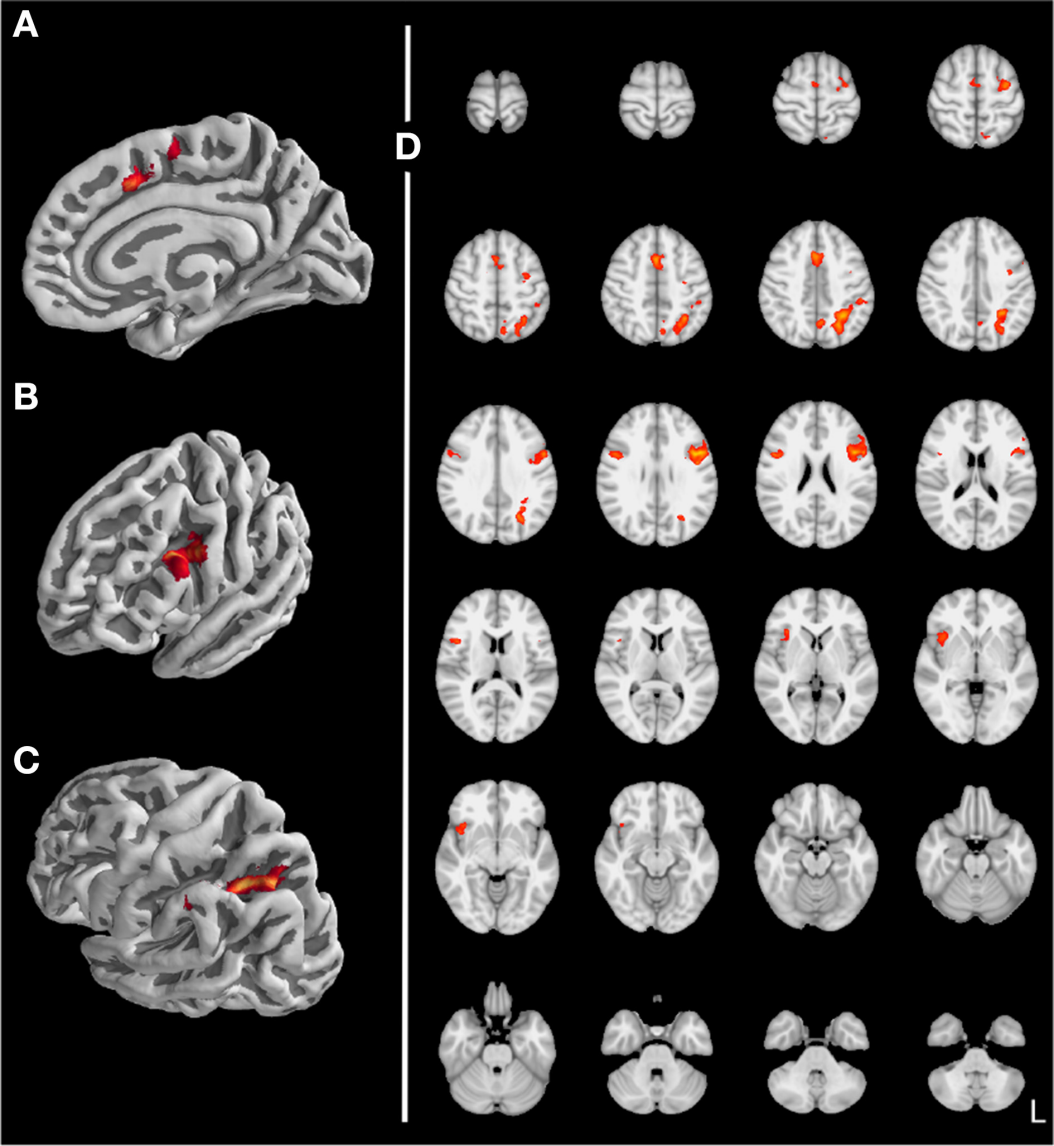
**Stroop task: Incompatible vs. compatible trials (*z* > 3.1, *p* < 0.05 with clustersize > 878 mm^3^, (A) paracingulate gyrus, (B) inferior frontal junction, (C) superior parietal lobule, (D) axial slices)**.

Stroop Task. To validate our data we first tried to replicate results found in the Stroop literature. When contrasting incompatible with compatible trials (see Table 2 and Figure 5), we found increased activity for the posterior medial frontal gyrus (pMFG; Figure 5A), inferior frontal gyrus (IFG; Figure 5B), and superior parietal cortex (Figure 5C), consistent with previous studies (e.g., MacLeod and MacDonald, 2000; Laird et al., 2005; Nee et al., 2007).

**Table 2:** **Stroop task: Incompatible vs. compatible trials (*z* > 3.1, *p* < 0.05 with clustersize > 878 mm^3^)**.

	**Volume**	**Z_max_**	**x**	**y**	**z**
**RIGHT HEMISPHERE**					
Insular cortex	1779	3.83	42	13	−5
Precentral gyrus	2036	3.98	48	8	22
**LEFT HEMISPHERE**					
Inferior frontal gyrus	5698	5.06	−44	9	6
Precentral gyrus	1433	4.27	−32	−8	56
Superior parietal lobule	7146	4.59	−30	−51	42
**MIDLINE**					
Paracingulate gyrus	3030	4.4	1	12	48
Precuneus cortex	1290	3.88	−4	−60	44

Region of Interest Analyses. Most important for our research question, we analyzed the activation pattern in early visual brain areas during performance of the Stroop task to examine modulation of sensory representations. While we were able to replicate previous studies in our whole brain analysis, whole brain analyses are in general considered often not to be sensitive enough to reveal modulation of small regions of interest for which additionally high inter-individual variability in exact anatomical location exists.

Percent signal change was calculated for V4 and V4α (i.e., color processing ROIs) and VWFA (i.e., word processing ROI) for each participant to examine whether activity in task-specific sensory areas shows enhancement and/or suppression as a function of conflict of the preceding trial.

**Figure 6:**
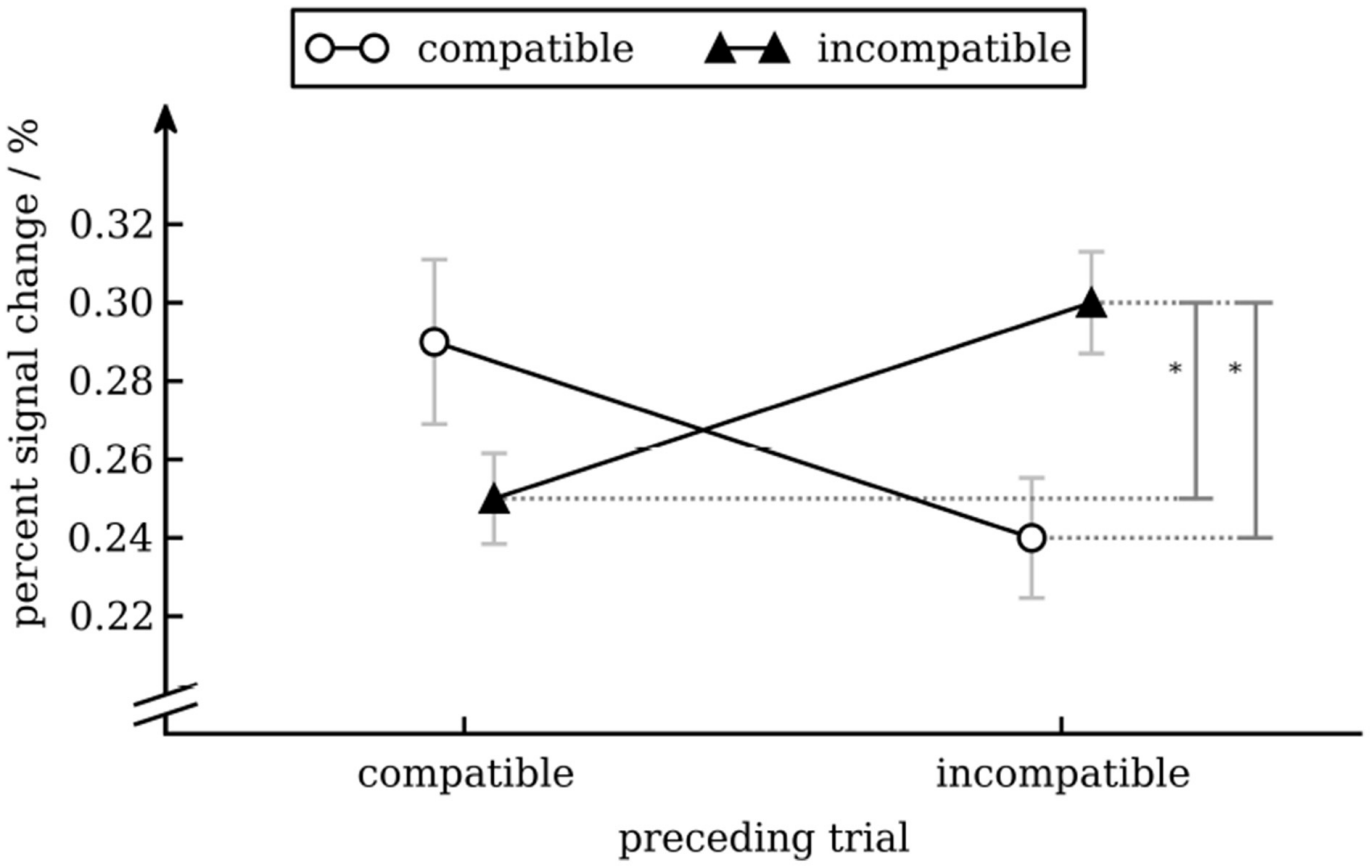
**Percent signal change in V4/V4α as a function of conflict in the previous and current trial**. Error bars show standard error of the mean (Cousineau and O'Brien, 2014).

To analyze activity in color processing ROIs we calculated a repeated measures ANOVA with percent signal change as dependent variable and conflict in the preceding trial (incompatible vs. compatible), conflict in the current trial (incompatible vs. compatible), hemisphere (left vs. right), position (anterior [V4α] vs. posterior [V4]) as independent variables for the four color processing ROIs. Except for the interaction-effect of conflict in the previous and conflict in the current trial [*F*_(1, 17)_ = 6.07, *p* = 0.025] none of the effects were statistically significant. We therefore pooled data of all four color ROIs for subsequent analyses. Separate *t*-tests showed that there was greater activity for incompatible trials that were preceded by an incompatible trial compared to those that were preceded by a compatible trial [*t*_(17)_ = 2.51, *p* = 0.022] and greater activity for incompatible trials compared to compatible trials when preceded by an incompatible trial [*t*_(17)_ = 2.67, *p* = 0.016] but not when preceded by a compatible trial (*p* = 0.18). No other effects were significant (see Figure 6). As a side note, it is interesting not to find an effect for position, i.e., no difference between V4 and V4α. Activation of V4 has been reliably found in passive viewing of color stimuli, activation of V4α is reliable seen in tasks requiring active manipulation of color information (Beauchamp et al., 1999). In another fMRI study V4α but not V4 showed activation during memory retrieval for color stimuli (Slotnick, 2009). Therefore, one might have suspected to find a stronger effect for V4α than for V4.

**Figure 7:**
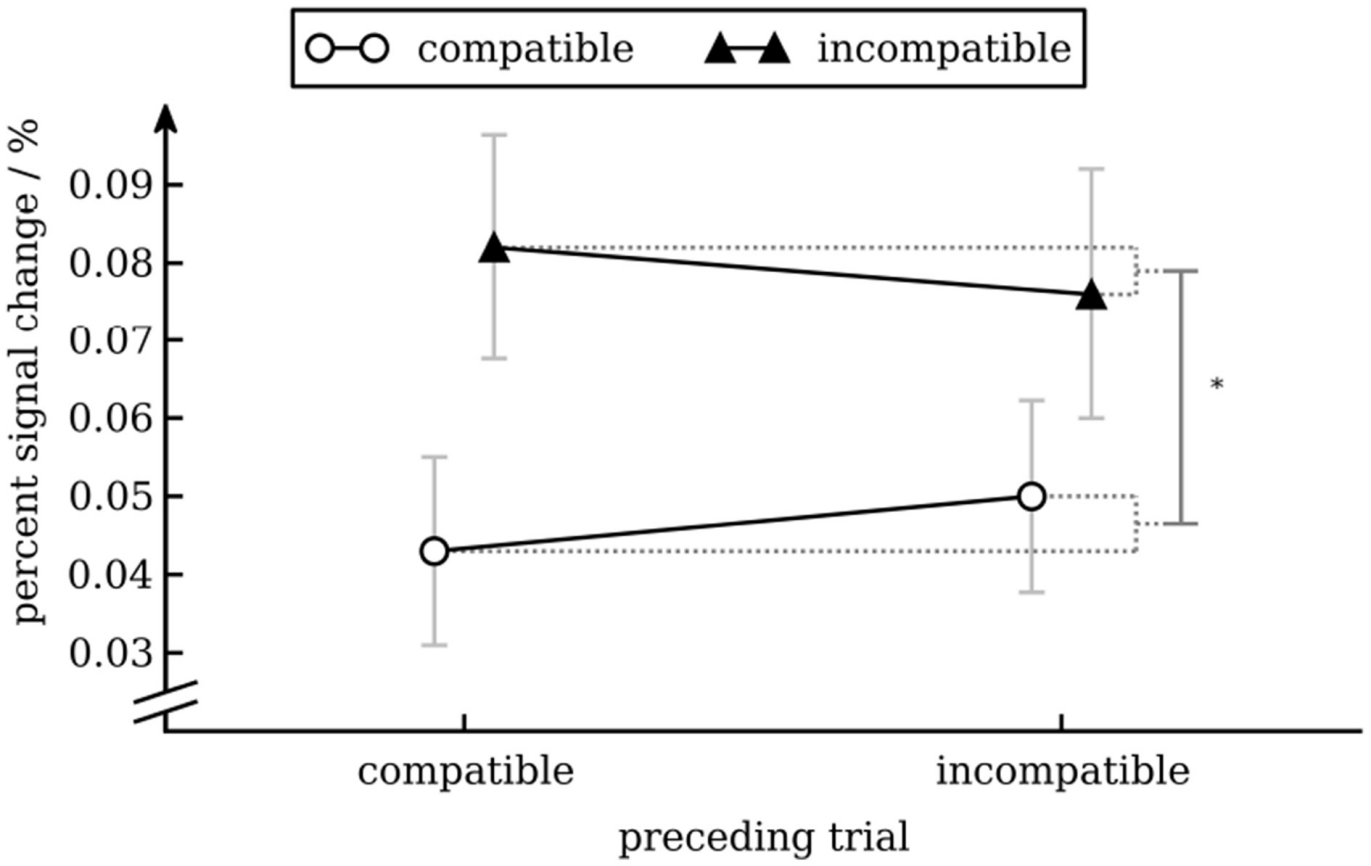
**Percent signal change in the VWFA as a function of conflict in the previous and current trial**. Error bars show standard error of the mean (Cousineau and O'Brien, 2014).

To analyze activity of the word processing ROI we calculated a repeated measures ANOVA with percent signal change as dependent variable and conflict in the preceding trial (incompatible vs. compatible) and conflict in the current trial (incompatible vs. compatible) as independent variables. Except for the main effect of conflict in the current trial [*F*_(1, 17)_ = 9.45, *p* = 0.007], reflecting that BOLD activity was greater for incompatible trials than for compatible trials, none of the effects were statistically significant (see Figure 7).

## 4. Discussion

Selectivity of information processing and adaptation thereof are key cognitive abilities for successful behavior in everyday life. To study these abilities in the laboratory, often so called interference tasks are used. Participants in these experiments are thought to enter a task-dependent cognitive set that is maintained for the duration of the task (Logan and Gordon, 2001), while information processing is fine-tuned on a short temporal scale to optimize task performance (Gratton et al., 1992). In this study, we aimed at specifying the neural mechanisms underlying this fine-tuning. Participants performed the Stroop task while BOLD signal was measured with fMRI. To exclude non-attentional accounts of the conflict recency effect (Hommel, 2004) we carefully controlled stimulus sequences. As in a recent study, we used an extended set of stimuli and responses to deconfound non-attentional sequential effects and conflict adaptation (Wendt et al., 2014).

Behaviorally, we found a Stroop effect in response times which was modulated by recent conflict. In functional imaging we found increased activity in a fronto-parietal network for incompatible trials compared to compatible trials was observed, consistent with the literature (e.g., MacLeod and MacDonald, 2000; Laird et al., 2005; Nee et al., 2007). As these regions have been found to be activated also by other interference paradigms than the Stroop task, they are thought to be involved more generally in the detection and resolution of cognitive conflict (Wager et al., 2005; Nee et al., 2007). It has been shown, that these areas can adaptively represent task-relevant information (adaptive coding; see Woolgar et al., 2011). Most importantly with respect to our research question, we found modulation by recent conflict of early sensory processing. This modulation was found in V4, an area that supports color processing but not in the VWFA, an area that support word processing.

We found that BOLD activity in V4 was higher for incompatible trials than for compatible trials when the previous trial was incompatible but not when the previous trial was compatible. This interaction effect is interesting, as from a simple enhancement model (upregulation of V4 after an incompatible trial) one would expect an equal increase in BOLD activity for compatible and incompatible trials (i.e., a main effect of conflict in the previous trial). Our results show that activity in V4 can be modulated by conflict in the current trial (likely through top-town control after the cognitive system has identified the compatibility level of the current trial). In our study this modulation occurs in a state of heightened cognitive control (i.e., after an incompatible trial) only. One interesting aspect of our data is that BOLD activity in V4 for cC trials was as high as for iI trials (see Figure 6). How this pattern evolves remains unclear. Nevertheless, this pattern has also been observed in another study (Egner and Hirsch, 2005), demonstrating its robustness. Importantly, activity for incompatible trials was greater after an incompatible trial than after a compatible trial, replicating results from aforementioned study (cp. Egner and Hirsch, 2005, Figure 2D). With respect to modulation of processing of task-irrelevant information, if anything one would expect decreased activity in the VWFA for incompatible trials. Interestingly, we actually observed increased activity for incompatible trials. Given that activity in VWFA was not modulated by conflict in the previous trial, the main effect of conflict in the current trial might simply reflect a time-on-task effect: As response times for incompatible trials were longer than for compatible trials, the VWFA was used for a longer period of time on these trials. Therefore, this effect might not reflect attentional modulation specific to incompatible trials (Weissman and Carp, 2013). Note that the same argument cannot be made for V4: there was no main effect of trial compatibility, but an interaction between current and previous trial compatibility (compare the figure for response times and percent signal change in V4).

Current models of cognitive control account for conflict adaptation effects by assuming attentional adjustment, i.e., fine tuning of selectivity of information processing, in response to cognitive conflict. One account supposes that the main mechanism of conflict adaptation involves adjustment of the response threshold (Gratton et al., 1992). In this model the cognitive system can give a response during an early (parallel) phase or a later (focused) phase in information processing. While during the parallel phase the cognitive system cannot distinguish between task-relevant and task-irrelevant information, during the focused phase it can. Giving a response during the parallel phase will lead to fast and correct responses for compatible trials and fast but wrong responses for incompatible trials, as it is assumed that the task-irrelevant information has a processing advantage during this phase. Giving a response during the focused phase on the other hand will lead to slower but correct responses for both compatible and incompatible trials. During the parallel phase only because of interruption of motor response execution and reprogramming of the motor response can a correct response be given for incompatible trials, which slows down response times substantially for such trials and puts them even at a disadvantage compared to responses given during the focused phase, for which this interruption and reprogramming is not needed. Conflict adaptation then is thought about as switching from giving the response during the parallel phase to giving the response during the focused phase. While adjustments might occur at the response level (see also King et al., 2010), given our data we have to state that modulation of sensory processing seems to be important too and is not included in this model. Another account assumes that adaptation to recent conflict is realized by enhancement of processing of task-relevant information early in information processing. This parallel distributed processing (PDP) model of the Stroop task (Cohen et al., 1990) proposes input units, that process task-relevant and task-irrelevant sensory information, response units, that plan and execute motor responses, and task demand units, that represent task rules and bias input units for successful task performance. A specific task-set (e.g., color-naming) is implemented by enhancement of processing of task-relevant information. The model has been extended (Botvinick et al., 2001) to account for adaptation to recent (Gratton et al., 1992) and frequent (Logan and Zbrodoff, 1979) conflict by adding a conflict monitoring unit. Concurrent activation of response units is used as a measure of response conflict and continuously signaled to task-demand units which in turn enhance processing of task-relevant information (i.e., increase selectivity of information processing). In this way, the current task-set is strengthened after an incompatible trial (recency effect) and this effect adds up, when incompatible trials occur often (frequency effect). More specifically, in this model the task-demand units increase activity of task-relevant input units when conflict is high, leading to reduced interference in the following trial and when conflict is frequent.

It is important to note that in this PDP model cognitive control could be implemented in various ways: At the input level cognitive control could act by activation of units that process task-relevant information, inhibition of units that process task-irrelevant information, or both. Furthermore, at the output level attention could modulate input signals of the response system by altering the connection weights between the input and output units or changing the output units' baseline activity (cp. Gratton et al., 1992), or a mixture of these mechanisms. While in the Cohen et al. model (1990) a task-set is implemented by enhancement of processing of task-relevant information, it is important to note that in other psychological models successful task performance in the Stroop task is often thought to result solely from suppression of task-irrelevant information. For instance, the greater Stroop effect in older adults (e.g., Logan, 1980; West and Alain, 2000; Langenecker et al., 2004) and in patients with schizophrenia (e.g., Henik et al., 2002; Henik and Salo, 2004) is proposed to result from a decline in the ability to inhibit processing of irrelevant sensory input (e.g., Cohn et al., 1984; Dulaney and Rogers, 1994). Similarly, deficits in inhibitory functions was proposed to underlie working memory impairment in the elderly (Gazzaley et al., 2005). Whether inhibition is one of the mechanisms of cognitive control is currently still highly debated. It is important to note that while we did not find evidence of suppression of early visual processing of task-irrelevant information, we cannot exclude that other areas involved in processing task-irrelevant information (e.g., higher-level language areas) have been suppressed.

It might be of value at this point to shortly review some cognitive neuroscience studies on task-set implementation, adaptation to recent conflict and adaptation to frequent conflict, because in the PDP model all should involve the same mechanism(s). Studies on post-error adjustments seem also related, as these adjustments can be seen as another instance of trial-by-trial adaptation.

A limited number of studies have examined activation of brain areas involved in processing of the task-relevant and task-irrelevant stimulus dimensions in task-set implementation in the Stroop and Stroop-like tasks. In some early functional imaging studies on the Stroop task, regional cerebral blood flow (rCBF) was measured using positron emission tomography while participants performed the classical Stroop task. These studies found either no evidence for enhancement of visual brain areas involved in color processing or suppression of left-hemisphere visual brain areas involved in word form processing (Pardo et al., 1990; despite the fact that they specifically examined these areas), suppression in the extrastriate cortex (Bench et al., 1993) or an increase in rCBF in the left lingual gyrus and a decrease in left lateral extra-striate cortex (Carter et al., 1995). Importantly, none of these studies localized visual color and word processing areas independently of the main task. In a more recent fMRI study, participants had to perform blocks of a Stroop task, with all stimuli being incompatible in some blocks and neutral in other blocks (Polk et al., 2008). For incompatible compared to neutral Stroop blocks they found increased activity in V4 and decreased activity in the VWFA and followed that both enhancement of processing of color information and suppression of processing of word information is part of task-set implementation. Taken together these studies suggest, that modulation of early visual areas might play a role in Stroop task performance and that this modulation might have the character of enhancement and/or suppression.

Given that all these studies on task-set implementation used block designs, none can separate transient (i.e., trial-by-trial) effects from sustained (i.e., block) effects. Therefore, it might be that the reported enhancement or suppression (Polk et al., 2008) are due to trial-by-trial conflict adaptation effects, as incompatible trials that followed incompatible trials occurred in incompatible Stroop blocks only. Given the results of our study, only enhancement effects can be explained by trial-by-trial effects. The inhibitory effects then might be specific to a sustained configuration of the cognitive system.

An fMRI study on adaptation to recent conflict explicitly tested if this effect is mediated by enhancement of processing of task-relevant information, suppression of processing of task-irrelevant information, or by both (Egner and Hirsch, 2005). In this fMRI study a face-word Stroop task was used. In some blocks participants had to respond to the faces, in others they had respond to the words. Behaviorally, they found interference and conflict adaptation effects under both task-sets. For incompatible trials following incompatible trials enhanced activity in the FFA was found, if faces were the task-relevant information, but not when faces represented the task-irrelevant information. They concluded that enhancement of task-relevant sensory information processing is the primary mechanisms of conflict resolution. Nevertheless, the results have to be interpreted carefully. In contrast to the classical Stroop task, where there is only one stimulus (with two stimulus dimensions), in the face-name Stroop task there are two stimuli. It is known that interference effects under such conditions are smaller (MacLeod, 1991), pointing to differences in the neurocognitive mechanisms between Stroop-like tasks using integrated and non-integrated stimuli. If effects found in one task generalizes to the other is currently not known. Additionally, in the experimental design they used, participants had to switch between face discrimination and word discrimination in a block-wise fashion. This manipulation was necessary to be able to examine activity in the FFA under two conditions, when faces were task-relevant and task-irrelevant, respectively. While a reverse Stroop-effect has been described, observing interference effects under both task-sets is unusual. It is known that switching between two task-sets in a block-wise fashion can lead to carry-over effects from one block to another (Allport et al., 1994; Allport and Wylie, 1999; Monsell, 2003). This task-set inertia could possibly explain the observed behavioral results, nevertheless, how task-set inertia effects interact with conflict adaptation effects is currently not well understood. Importantly, our results show that the results of this study generalize to the classical Stroop task without task-switching. Therefore, our results underline the notion that enhancement of processing of task-relevant information is involved in adaptation to recent conflict, while there is no evidence so far for suppression of processing of task-irrelevant information.

In an ERP study on adaptation to frequent conflict, no modulation of early sensory components by conflict frequency has been found (Purmann et al., 2011). In this study, frequent conflict was associated with reduced flanker interference in response times and error rate. The amplitude of the fronto-central N2 was larger and latency of the central P3 longer for incompatible stimuli and both effects were smaller when conflict was frequent. Most interestingly, neither amplitude nor latency of the posterior P1, as index of early visual processing, was modulated by conflict frequency, suggesting that adaptation to frequent conflict is not mediated by enhancement or suppression of sensory information processing but by adjustments at a later stage of information proessesing.

Finally, in a study on post-error adjustments in the Eriksen flanker task, post-error slowing has been found and that BOLD activity for correct trials following errors compared to correct trials following correct trials resembles the activated network during motor inhibition (Marco-Pallares et al., 2008). As noted above, adjustment of the response threshold might also play a role in conflict adaptation (Gratton et al., 1992). Similarly, in another study on post-error adjustments using a face Simon task, post-error slowing was accompanied by BOLD activation of a comparable network and suppression of somatomotor cortex, and post-error reduction of interference was accompanied by enhancement of activity in the FFA (King et al., 2010), showing that both, enhancement of task-relevant information and adjustment of the response threshold are mechanisms recruited in response to error trials.

Although it is plausible (and most parsimonious) to assume that the mechanisms underlying implementation of a task-set, adaptation to recent conflict, adaptation to frequent conflict, and post-error adjustments are identical, there is no clear evidence for this assumption so far. Quite to the contrary, evidence raising doubt on this assumption is accumulating. Additionally to the similarities and differences already mentioned, it has been found in behavioral studies that while frequency effects can be shown in an early phase of motor responses (i.e., movement initiation), recency effects seem to be confined to later phases (Purmann et al., 2009), recency effects disappeared after controlling for feature integration effects, frequency effects were still present (Fernandez-Duque and Knight, 2008), and that while recency effects vanished over the course of the experiment, frequency effects can be found even at the end of the experiment (Mayr and Awh, 2009). Our data further calls the assumption of a “one-fits-all” mechanism into question: If adaptation to recent conflict is implemented by strengthening the current task-set, then studies on task-set implementation should not show suppression of processing of task-irrelevant information. Therefore, the discrepancy of studies on task-set implementation and studies on adaptation to recent conflict suggests that the underlying mechanisms might differ.

To summarize, we observed that activity for incompatible trials following incompatible trials was increased in V4 (Bartels and Zeki, 2000) while activity in the VWFA (Cohen et al., 2000) was not modulated by conflict level in the previous trial. We thereby replicated studies that used a face-word Stroop task. As we carefully controlled for non-attentional sequential effects we argue that the trial-by-trial effect we found represent attentional modulation proper and that our brain imaging results specify the interactions of the task demand units with the input units in the PDP model (Cohen et al., 1990; Botvinick et al., 2001). We conclude that adaptation to recent conflict in Stroop-like tasks (color-word Stroop task, face-word Stroop task) seems to mainly involve enhancement of task-relevant information but not suppression of task-irrelevant information and that this mechanism might differ from the mechanisms underlying other instances of cognitive control.

## Author contributions

The statement about the authors and contributors can be up to several sentences long, describing the tasks of individual authors referred to by their initials and should be included at the end of the manuscript before the References section.

### Conflict of interest statement

The authors declare that the research was conducted in the absence of any commercial or financial relationships that could be construed as a potential conflict of interest.
